# Chronic Diseases Self-Efficacy Profiles and Healthcare Utilisation Patterns Among Older Adults with Multimorbidity in Albania: A Person-Centred Latent Profile Analysis

**DOI:** 10.3390/nursrep16100367

**Published:** 2026-10-16

**Authors:** Velide Pulomemoj, Anisa Subashi, Arshad Mehmood, Lorena Serjanaj, Fatjona Kamberi

**Affiliations:** 1 Department of Biomedicine and Prevention, University of Rome Tor Vergata, 00133 Rome, Italy; 2 Faculty of Social Sciences, University of Tirana, 1010 Tirana, Albania; anisasubashi@gmail.com; 3 Department of Health and Social Care, CECOS College, London E1 4NQ, UK; arshad@cecos.ac.uk (A.M.); lorena.tutor@cecos.ac.uk (L.S.); 4 Scientific Research Center for Public Health, Faculty of Health, University of Vlore “Ismail Qemali”, 9401 Vlora, Albania; fatjona.kamberi@univlora.edu.al

**Keywords:** nursing, chronic-disease self-efficacy, older adults, latent profile analysis, healthcare utilisation patterns, primary healthcare, Albania

## Abstract

**Background/Objectives**: Multimorbidity renders self-efficacy a continuing partnership between older people, families, and healthcare and social care services. We examined whether nine domain-specific self-efficacy indicators were best understood as one overall level of confidence or as recurring configurations in which confidence was distributed differently across everyday tasks. The primary aim was to identify and interpret these configurations in older adults attending primary care in Vlora Municipality, Albania. Secondary aims were to evaluate the quality and reproducibility of the solution and to describe health literacy and six-month healthcare utilisation, without treating either as a cause or as a profile-defining variable. **Methods**: This verified secondary cross-sectional analysis used data from 464 community-dwelling adults aged 65 years or older with at least two chronic conditions recruited through five primary healthcare centres. After two questionnaire values were checked against the source records, 459 participants with complete nine-domain self-efficacy data formed the primary sample. We compared 40 Gaussian finite-mixture candidates: one to ten profiles under four covariance structures. Model choice used a combination of Bayesian information criterion (BIC), integrated completed likelihood (ICL), Akaike information criterion (AIC), entropy, average posterior probability, profile size, convergence, covariance diagnostics, interpretability, and reproducibility across 50 random starts. There is no single closed-form power calculation for profile enumeration; therefore, precision was assessed empirically with missing-item, domain-omission, and bootstrap checks. Health literacy and healthcare utilisation were kept outside profile formation and were described with conditional adjusted count models. **Results**: The retained exploratory Profile-specific full-covariance model (VVV) two-profile solution had 249 and 210 participants (mclust BIC = −8125.080; ICL = −8181.987; AIC = 7675.013). Assignment quality was reasonably precise (entropy = 0.814; average posterior probabilities = 0.961 and 0.931), but the profile solution was not simply a low-to-high severity scale. One pattern was stronger for finding information, obtaining support, and communicating with physicians; the other was stronger for exercise, household and social activities, symptom management, and emotional management. In 100 bootstrap refits, the adjusted Rand index ranged from 0.212 to 0.865 (median 0.584), so stability was moderate. After covariate adjustment, Profile 2 had a modelled general-practitioner visit rate that was 19% lower than Profile 1 [incidence rate ratio (IRR) = 0.809; 95% confidence interval (CI) 0.687–0.953; *p*-value = 0.011]; this is an exploratory association conditional on the fitted model, not evidence of a causal effect. **Conclusions**: A profile can show how confidence is organised across daily self-efficacy tasks even when two groups have similar overall means. For health and social care, the findings support assessment that links information and communication with activity, symptom management, emotional wellbeing, family support, and community resources. The profiles are exploratory conversation prompts rather than diagnostic categories, and the cross-sectional data do not show that self-efficacy causes healthcare use.

## 1. Introduction

Living with two or more long-term conditions changes the texture of everyday life. Medicines, appointments, diet, movement, symptom monitoring, and advice from several professionals must be fitted around pain, fatigue, sensory change, reduced mobility, and family responsibilities. Although services are often organised around separate diagnoses, the person experiences one connected workload. Multimorbidity is therefore a clinical problem and a practical challenge for health and social care [1,2].

Much of chronic-illness management happens between appointments. Older individuals have to notice changes, decide what to do, follow treatment, adapt routines, and recognise when professional help is needed. Self-efficacy is a person’s confidence in their ability to organise and carry out these actions in a particular situation [3]. It is not the same as knowing what to do, wanting to do it, having health literacy, or having the physical ability and resources to do it. For nurses, family doctors, rehabilitation teams, community workers, and social-care staff, the useful question is therefore not simply whether a person feels confident but which part of daily self-management is hardest [4].

As conditions accumulate, treatment plans become harder to coordinate, symptoms may not have one clear source, and advice from different services may not fit together. These pressures help explain why self-management programmes may have different effects for different people and settings [5,6,7]. They also make task-specific confidence important. Someone may feel able to ask a clinician for information yet feel less able to exercise with pain or breathlessness, respond to changing symptoms, or maintain social activity. A single total score can hide these differences [8,9].

Confidence is shaped by the circumstances in which self-efficacy takes place. Financial pressure, loneliness, fear of falling, treatment burden, and poorly coordinated services can make an advised behaviour difficult even when the person understands the advice [10,11,12,13,14]. Family, professional, and community support can make the same action more possible. A lower domain score may therefore point to a skill that needs practice, but it may also signal unresolved symptoms, inaccessible services, or a care plan that does not fit the person’s life. Clear communication with clinicians is one part of this wider patient-centred environment [15].

Health literacy can make health information easier to use, but information alone does not guarantee action. It includes being able to find, understand, judge, and use information about health, and limited health literacy is common among people living with multimorbidity [16]. Longitudinal research has linked health literacy with later self-efficacy and preventive care use [17]. In the parent Albanian cohort, health literacy was positively associated with chronic disease self-efficacy in a variable-centred analysis [18]. The present study extends that study by asking whether people with similar overall confidence organise that confidence differently across everyday tasks. This distinction is relevant to support for disadvantaged older adults and to services that treat social connection as part of self-management [19,20].

Self-efficacy, patient activation, and healthcare utilisation overlap, but they answer different questions. Patient activation combines knowledge, confidence, and skills for managing health, and it has been associated with hospital and emergency department uses [21,22,23]. These associations are observational and vary between settings; activation is also broader than confidence in a particular task. The association can work in the opposite direction as well: worsening illness, frailty, or previous hospital contact may reduce confidence while increasing service use [24,25]. Healthcare contact is therefore not a simple measure of success. Fewer visits may reflect good support, while more primary care or emergency contacts may reflect appropriate monitoring or greater clinical need [26,27,28,29].

Latent profile analysis offers a complementary person-centred description. In simple terms, it looks for recurring combinations of answers across several domains instead of reducing everyone to one average or imposing arbitrary cut-points [30,31,32]. In this paper, the words profile and pattern refer to these model-derived combinations; they do not imply a diagnosis or a permanent type of person. Recent studies have used profile methods to describe self-management behaviours in older adults with chronic disease and in people with comorbid diabetes and hypertension [33,34]. The resulting solution depends on which indicators are included, how their relationships are modelled, how many profiles are considered, and how large the sample is [30,31,32]. Its practical purpose is to reveal differences in support needs rather than to assign people to permanent categories.

The central question was whether variation in chronic disease self-efficacy reflected mainly a general level of confidence or recurring combinations of relative strengths and difficulties. If it were primarily a continuum, support would be expected to differ primarily in intensity. However, if confidence varied by task, care might need to focus on particular activities, symptom planning, emotional support, or help from family and community services. Framing the question in this way links the statistical analysis to the everyday work of nursing, primary care, rehabilitation, community health, and social care.

The present analysis extends the parent study [18], which examined the average relationship between health literacy and chronic disease self-efficacy, by examining how confidence is distributed across tasks within the same person. Health literacy and healthcare utilisation were kept external to the profile model such that they could be described after the self-efficacy patterns had been estimated, rather than helping to define the groups that were later compared. This separation follows person-centred guidance and protects the interpretation of the profiles as patterns of self-efficacy alone [30,31,32]. Because profile solutions can change with the indicators, covariance assumptions, candidate range, sample composition, and classification quality, the analysis was explicitly exploratory [30,31,32,35,36].

The aim was to identify and interpret configurations of chronic disease self-efficacy among older adults with multimorbidity receiving primary healthcare in Vlora Municipality, Albania. The objectives were as follows: (1) compare one- to ten-profile solutions across diagonal and full covariance structures; (2) evaluate relative fit, assignment quality, profile size, convergence, and covariance diagnostics; (3) determine whether the selected pattern was reproduced with alternative missing-item handling, domain omission, and bootstrap resampling; (4) distinguish a general confidence gradient from domain-specific shape using participant-centred scores; and (5) describe health literacy and six-month healthcare utilisation across profiles using adjusted, explicitly non-causal models. The resulting profiles were intended as working descriptions to support person-centred conversations and care planning, not as fixed, diagnostic, or causal categories.

## 2. Materials and Methods

### 2.1. Study Design and Participants

We analysed data from 464 community-dwelling adults aged 65 years or older with at least two diagnosed chronic conditions. After two questionnaire values were verified against the source records, 459 participants with complete data across all nine self-efficacy domains constituted the primary sample. Participants were recruited through five primary healthcare centres in Vlora Municipality, Albania. Participant recruitment was conducted over a six-month period, from February to July 2025. Eligible participants were randomly selected from the family physician registries of the participating healthcare centres. Eligibility and the presence of ≥2 chronic conditions were verified using patients’ medical records and family physician registries. Based on the sample size calculation of the parent study, a minimum of 384 participants was required [18]. Selected patients were informed about this study’s purpose and procedures, and those who provided written informed consent were recruited until the required sample size was achieved. The exact number of potentially eligible patients and those initially approached was not recorded. Individuals with cognitive impairment that prevented informed consent or reliable questionnaire completion were excluded. The sampling frame therefore reflects older adults reached through primary care, rather than all older residents or people who do not seek healthcare. The reporting followed the Strengthening the Reporting of Observational Studies in Epidemiology (STROBE) guidelines [37].

### 2.2. Measures

Chronic disease self-efficacy was assessed with Stanford Chronic Disease Self-Efficacy Scale (CDSES-33) items scored from 1 (not at all confident) to 10 (totally confident) [38]. The 33-item instrument was translated and culturally adapted into Albanian through independent forward and back-translation by bilingual experts, followed by pilot testing with native Albanian speakers to assess clarity and cultural appropriateness. Based on participant feedback, the instrument was revised and proofread before finalisation. The overall CDSES-33 instrument demonstrated excellent internal consistency (Cronbach’s α = 0.974), with Cronbach’s α values for the individual domains ranging from 0.840 to 0.958, indicating good to excellent internal consistency across all multi-item domains [18]. The items covered nine everyday domains: exercise, obtaining health information, support, communication with physicians, disease management, household activities, social activities, symptom management, and emotional management. For a multi-item domain, the participant’s valid item responses were averaged; the single item on obtaining information retained its original score. The domain scores were therefore on a common 1-to-10 scale, but they do not measure health status, adherence, or quality of care. A higher score denotes greater reported confidence in that set of tasks. The total 33-item score was used only to examine whether the profiles followed an overall continuum; it was not entered as a tenth clustering indicator. No factor analysis was conducted in the Albanian cohort. Therefore, the nine-domain structure was adopted from the original source instrument and has not been independently validated in the Albanian population. Table 1 presents the domain distributions and internal consistency estimates.

Health literacy was measured with the 16-item European Health Literacy Survey Questionnaire (HLS-EU-Q16). We first applied the standard dichotomous scoring procedure. A strict 0–16 score was calculated only when all 16 answers were present [38,39]. For sensitivity analysis, a prorated score was calculated only when at least 14 answers were valid; the observed valid item proportion was then scaled to the 16-item range. An unanswered question is not equivalent to a negative response; thus, an incomplete questionnaire was not converted to a low score using ‘rowSums(..., na.rm = TRUE)’. Age; sex; education; body mass index; self-rated health; number of chronic diseases; health centre; residence; and six-month counts of general practitioner visits, emergency department visits, hospital admissions, and hospital nights were treated as external variables; none helped define the profiles.

### 2.3. Verification of Source Data and Handling of Missing Values

We checked the analysis dataset against the original questionnaires before any model was fitted. The source-checking ledger recorded two corrections: age 37 was confirmed as 67 for study Identification (ID) 269, and height 72 was confirmed as 172 for study ID 310. These changes were verified from the source records and were not produced by an automatic range-replacement rule. The parent sample remained 464. The strict primary analysis included 459 participants with complete responses across all nine self-efficacy domains, while available-item scoring retained 463 participants. Strict HLS-EU-Q16 scores were available for 462 participants, and no body mass index (BMI) value was missing in the verified parent sample. No item-level imputation was performed. The full sample flow is shown in the Supplementary Materials Table S1.

### 2.4. Latent Profile Modelling

Latent profile analysis (LPA) treats the observed sample as a mixture of unobserved groups with members who have similar average patterns across the indicators [30,31,32]. We standardised the nine domain scores using the sample mean and standard deviation such that the fitted model compared relative domain patterns rather than allowing a domain’s measurement scale to dominate the solution. Gaussian finite-mixture models were fitted in R 4.3.3 using mclust 6.1.3 [31,39]. The rerun examined one through ten profiles (G = 1–10) under four covariance structures, yielding 40 candidate models. The range was deliberately extended beyond five profiles such that a solution could be judged against higher-profile alternatives. The equal diagonal covariance model (EEI) and profile-specific diagonal covariance model (VVI) represent diagonal covariance models: they assume that domains are unrelated within a profile after conditioning on profile membership, with the EEI model imposing a common diagonal form and the VVI model allowing profile-specific diagonal covariances. The equal full covariance model (EEE) and profile-specific full covariance model (VVV) represent full covariance models that allow domains to remain related within a profile: the EEE model uses a common covariance form, and the VVV model allows the covariance matrix to vary by profile. These four structures provide a transparent comparison of local independence versus within-profile dependence and equal versus profile-specific covariance. They do not represent every possible mclust parameterisation; thus, the covariance grid is itself a modelling choice and is interpreted as a sensitivity framework rather than an exhaustive search [30,31,32,35,36].

For each candidate, the default fit was supplemented by 50 random starts, providing up to 51 attempted fits per candidate and up to 2040 attempted fits across the 40-model grid [31]. Multiple starts reduce the chance that the reported solution is only a local maximum of the likelihood. Model comparisons considered AIC and BIC as relative fit criteria, ICL as a fit criterion that also penalises overlapping classification, entropy and average posterior probability as assignment diagnostics, profile size, convergence, covariance eigenvalues and condition numbers, parsimony, and whether the resulting pattern could be explained in a meaningful health and social care context [30,31,32,35,36]. Larger (less negative) mclust BIC values indicate better relative fit; none of these indices alone proves that a latent group is a natural or permanent entity. The full fit and start records are supplied in Supplementary Materials Figure S1 and Table S2, respectively. Participants were assigned to the profile with the largest posterior probability for descriptive and conditional external analyses. For each participant, the assigned profile was the one with the highest posterior membership probability. This probability expresses the model’s uncertainty about profile membership; it is not a diagnosis, and the labels in Profiles 1 and 2 do not represent clinical cut-offs.

### 2.5. Precision, Power, and Safeguards for Model Choice

A conventional power calculation was not specified because there is no single power formula for deciding the number of profiles in an LPA. Simulations show that recovery of the correct solution depends on several features at once, including sample size, the number and quality of indicators, separation between profiles, profile prevalence, covariance and distributional assumptions, and the model-selection rule [35,36]. With 459 complete cases and nine indicators, the data can describe broad, well-represented patterns, but they cannot guarantee the correct number of profiles or stable estimates for very small groups. We therefore reported empirical checks instead of a post hoc power percentage: the analysis covered 40 candidates, used up to 51 starts for each candidate, and assessed the selected model through profile sizes, entropy, posterior probabilities, eigenvalue diagnostics, missing-item and omission analyses, and 100 bootstrap refits. These checks describe the precision and reproducibility of the observed solution; they do not convert an exploratory mixture into a confirmed population taxonomy.

### 2.6. Robustness and Continuum Diagnostics

We checked whether the result depended on incomplete domain responses or on any single indicator. We repeated the complete search in the available-item sample and compared assignments for participants present in both analyses using the adjusted Rand index (ARI) and ordered agreement. The ARI corrects the agreement between two classifications for agreement expected by chance; ordered agreement additionally asks whether the same participants remain in the same relative profile order. We repeated the strict search after omitting each domain in turn. Finally, we drew 100 bootstrap samples from the strict primary data, refitted the selected model in each sample, and compared the refitted classifications of the original participants using the ARI. These are reproducibility checks rather than evidence that the profiles are permanent or naturally occurring population types [30,31,32,35,36].

To distinguish overall confidence from the way confidence was distributed, we calculated Pearson and Spearman correlations between the numeric profile label and the total 33-item score. We also centred the nine domains within each participant such that each value represented how far a domain was above or below that person’s own nine-domain average. Positive values mean that a domain was relatively stronger for that participant; negative values mean that it was relatively weaker. This participant-centred representation answers whether the profiles mainly separate individuals who feel confident from those who feel less confident or whether they show different combinations of everyday strengths and difficulties. Because the order of neutral profile labels is arbitrary, the near-zero magnitude of the correlation, not its sign, is the meaningful result [30,31,32].

### 2.7. External Health Literacy and Healthcare Utilisation Analyses

Health literacy and healthcare utilisation were kept outside the profile model. This separation prevents the outcomes of interest from helping to define the groups that are later compared and follows recommended person-centred workflows [30,31,32]. Strict and prorated health literacy scores (HLSs) were calculated according to the European Health Literacy Survey framework [40,41] and summarised with posterior weights as descriptive external means. We examined six-month general practitioner visits, emergency department visits, hospital admissions, and hospital nights with negative binomial count models, which allow the variance of utilisation counts to exceed their mean. All participants had the same reported six-month observation window; thus, no exposure offset was added. The adjusted models included profile, age, sex, number of chronic diseases, self-rated health, education, residence, health centre, and BMI. Age and BMI were entered linearly in the primary models, with spline specifications examined as a nonlinear sensitivity analysis. Health centre was included as a fixed-effect covariate; cluster-robust standard errors were not estimated because only five centres were available. We checked whether the fitted models reproduced the observed proportion of people with no events; an additional zero-inflation component was not needed. Exponentiated profile coefficients are reported as incidence rate ratios (IRRs), the ratio of the modelled expected count for one profile to the reference profile. Modal assignment was used for transparent external comparisons, but it does not fully propagate classification uncertainty. Bias-adjusted three-step, maximum-likelihood, and Bolck–Croon–Hagenaars method for distal outcomes (BCH) approaches are available for distal outcomes [42,43,44]; the present results are therefore conditional descriptions rather than fully error-corrected effects. Healthcare utilisation reflects clinical need, monitoring, access, and organisation of care; thus, it was treated as an external care-use outcome rather than a direct measure of self-management success [21,22,23,24,25,26,27,28].

### 2.8. Ethical Considerations

The parent study was conducted in accordance with the Declaration of Helsinki and applicable Albanian data-protection requirements. Participation was voluntary; all participants provided written informed consent; and ethical approval was granted on 3 June 2024 (Approval No. 1084/1) by the Operator of Healthcare Services, the Regional Directorate of Vlora, and the Directorate of Public Health and Health Education [18]. The present secondary analysis used de-identified data covered by the original approval and did not require separate ethical approval.

## 3. Results

### 3.1. Analytical Sample and Domain Characteristics

The verified parent dataset contained 464 participants. The strict primary nine-domain sample contained 459 participants (98.9%), the available-item sensitivity sample contained 463, and strict HLS-EU-Q16 scores were available for 462. Mean domain scores ranged from 4.60 for exercise to 6.38 for communication with physicians on the 1-to-10 confidence scale. The full 33-item scale had high internal consistency (alpha = 0.974), and the alpha value for the eight multi-item domains ranged from 0.839 to 0.958. The nine domains were positively related (r = 0.403–0.792). Thus, a shared confidence signal was present, but the correlations were not so high that the domains became interchangeable. Taken together, these findings support examining the shared level of self-efficacy and the task-specific differences that may be important in care.

### 3.2. Expanded Profile Search and Selected Solution

The expanded search tested 40 candidates, covering one to ten profiles for each of the four covariance structures. In the strict primary sample, VVV_G2 had the most favourable BIC (mclust BIC = −8125.080), with AIC = 7675.013 and ICL = −8181.987. The next VVV solutions had lower BIC values (VVV_G3 = −8148.790; VVV_G4 = −8276.233), whereas EEE_G7 had BIC = −8480.912 and contained a profile of only five participants. VVV_G2 therefore combined the relative fit with two well-sized profiles rather than many very small groups. We interpreted the fit statistics together with assignment quality, profile size, convergence, covariance diagnostics, and substantive clarity. This does not make two profiles the unique or true answer; it indicates that they were the most proportionate summary among the models examined. The main candidates are shown in Table 2, with the full grid in Table S2 (Supplementary Materials).

The two-profile result was consequently retained as exploratory. Only one of the 51 starts yielded a warning-free numerical fit; the remaining starts reported singular-covariance warnings. The fitted covariance matrices were positive definite (minimum eigenvalues of 0.0316 and 0.2340), although their condition numbers (252.2 and 18.4) show that the first was relatively ill-conditioned. EEE_G7 converged cleanly but produced a five-person profile, showing why convergence alone cannot determine usefulness. Models with more profiles also increased complexity without giving a clearer care-relevant interpretation. We therefore present VVV_G2 as a transparent description of this sample and retain its numerical uncertainty in the interpretation.

### 3.3. Differences Between the Selected Profiles

**Figure 1:**
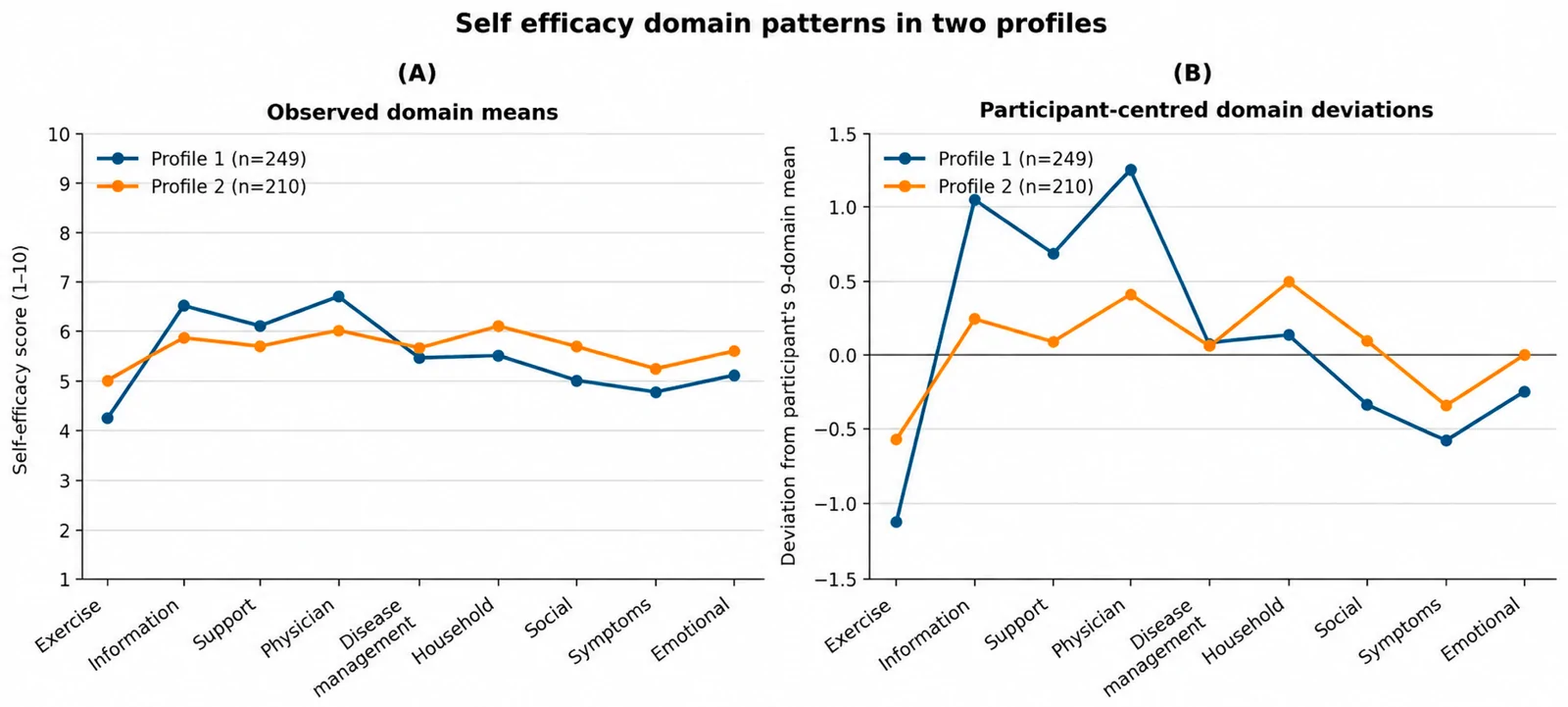
Self-efficacy domain patterns in the strict primary sample. Panel (**A**) shows observed domain means. Panel (**B**) shows each domain relative to the participant’s own nine–domain mean. Positive and negative values indicate relative elevation and attenuation, respectively; they are descriptive contrasts, not validated clinical strengths, deficits, or cut-points.

VVV_G2 assigned 249 participants to Profile 1 and 210 to Profile 2. The assignments were fairly clear, although not perfect: entropy was 0.814, and the average posterior probabilities were 0.961 and 0.931. The raw domain means did not follow a simple low-to-high ordering. Profile 1 was higher for obtaining information (6.53 versus 5.84), support (6.08 versus 5.68), and communication with physicians (6.70 versus 6.00). Profile 2 was higher for exercise (5.02 versus 4.24), household activities (6.08 versus 5.50), social activities (5.68 versus 5.03), symptom management (5.24 versus 4.79), and emotional management (5.59 versus 5.12); disease management scores were similar (5.63 versus 5.46). The overall nine-domain means were close (5.49 versus 5.64). Thus, the profiles differed in the balance of confidence across tasks rather than separating a generally confident group from a generally unconfident group. Panel A of Figure 1 shows these contrasts.

The participant-centred plot provides a second view of the same result. Once each person’s nine-domain average was removed, Profile 1 remained relatively higher for information, physician communication, and support but lower for exercise, social activities, symptoms, and emotional management. Profile 2 was flatter, with relative elevation in household activities, physician communication, information, and support and relative attenuation in exercise and symptoms. Correlations between the numeric profile label and the 33-item total were 0.063 (Pearson) and 0.065 (Spearman); because the labels are arbitrary, the size of these correlations is more informative than their sign.

Elevation explained 8.2% of the between-profile contrast, while shape explained 91.8%. The main distinction was therefore the distribution of confidence across tasks, not overall confidence. The profiles add detail to the total score, but they do not establish two clinically distinct groups.

### 3.4. Robustness and Sensitivity Checks

When the available-item analysis was compared with the strict analysis, VVV_G2 again had the best BIC. For the 458 participants included in both datasets, the ARI was 0.983, and ordered agreement was 0.996. VVV_G2 also led each single-domain omission analysis. These results suggest that the broad two-pattern description was not driven by one missing item or one particular domain. However, each omission grid produced only one clean start per candidate; thus, these analyses are reassuring but directional rather than definitive evidence about the importance of any individual domain.

All 100 bootstrap refits of VVV_G2 were completed without warnings. The adjusted Rand index against the original classifications ranged from 0.212 to 0.865 (median 0.584; mean 0.578), and the smallest bootstrap profile ranged from 87 to 229 participants (median 171). Higher adjusted Rand values mean greater agreement with the original assignments; the wide range shows that agreement varied across resamples. The selected model therefore reproduced a broad two-pattern summary in many resamples but not with the consistency expected of a sharply defined taxonomy. Together with the singular-covariance warnings in the random-start search, this supports using the profiles for exploratory description and discussion, not for clinical labelling or automatic decisions.

### 3.5. Health Literacy and Healthcare Utilisation Patterns

Posterior-weighted descriptive means for strict HLS-EU-Q16 scores were 10.41 for Profile 1 and 11.02 for Profile 2 on the 0–16 scale. The prorated sensitivity means were 10.42 and 11.03, respectively. The strict and prorated rules therefore led to the same substantive description: Profile 2 had a slightly higher mean, but the difference was small (see Table 3). These values describe how health literacy was distributed in the selected mixture; they do not show that health literacy determined profile membership or that changing health literacy support would move someone into another profile.

The fitted negative-binomial models reproduced the observed proportion of people with no visits or admissions; thus, the data did not require a separate model for an excess of zero-use observations (see Table 4). After adjustment for age, sex, chronic-disease count, self-rated health, education, residence, health centre, and BMI, Profile 2 had a modelled general-practitioner visit rate about 19% lower than Profile 1 [incidence rate ratio (IRR) = 0.809, 95% CI 0.687–0.953, *p* = 0.011]. The corresponding profile estimates for emergency-department visits, hospital admissions, and hospital nights were uncertain. Profile models also did not improve AIC over total-score models: general practitioner (GP) visits 1926.218 versus 1918.390; emergency department (ED) visits 1453.557 versus 1445.485; hospitalisations 913.747 versus 899.466; and hospital nights 1326.560 versus 1304.662. Spline sensitivity models improved AIC for GP visits, ED visits, and hospitalisations but not hospital nights; this changes the preferred functional form for some outcomes, not the cross-sectional interpretation. No formal multiplicity correction was applied; thus, the GP contrast should be treated as an exploratory signal rather than a confirmatory result. These estimates should not be interpreted as a simple scale from “good” to “bad” care: a contact may reflect unmet need, appropriate monitoring, continuity, or an acute problem.

## 4. Discussion

### 4.1. Principal Findings and Contribution

This study’s primary contribution is a person-centred view of chronic-disease self-efficacy among older adults living with multimorbidity in an Albanian primary care setting. The expanded analysis identified two broad configurations with similar overall confidence but different distributions of confidence across everyday tasks. One configuration was relatively stronger for finding information, receiving support, and communicating with physicians. The other was relatively stronger for exercise, household and social activities, symptom management, and emotional management. The result is therefore about the organisation of confidence, not about two fixed types of patients or a simple high-versus-low ranking.

This distinction is relevant to practice because a total score does not show which task is most difficult in daily life. Looking across domains gives the person and the care team a clearer starting point for discussion. Depending on the pattern, the next step might be teach-back and accessible information, supported exercise or rehabilitation, a medication or symptom review, or emotional and social support. The profile cannot make that decision on its own; it can, however, encourage the team to ask what is preventing action and which family, community, primary care, nursing, or social care resource might help.

The expanded grid, repeated random starts, covariance checks, missing-item analyses, domain-omission analyses, and bootstrap refitting render the selection easier to scrutinise than a single fitted model. They also identify the limits of the evidence. VVV_G2 was the most balanced candidate in this dataset, but its moderate bootstrap agreement and the numerical warnings mean that the pattern still requires replication. No conventional a priori power percentage was calculated: In LPA, the probability of recovering the correct number of profiles depends jointly on sample size, number of indicators, separation, profile prevalence, covariance assumptions, and the selection rule [35,36]. With 459 complete participants and nine indicators, this study could estimate broad, well-populated patterns, but it cannot guarantee correct enumeration or validate small profiles. The wider contribution is therefore methodological and care-oriented: it shows how a multidomain assessment can bring the everyday work of multimorbidity into view without turning a statistical profile into a diagnosis [30,31,32,33,34,35,36].

### 4.2. What the Domain Pattern Contributes

A person-centred analysis is useful only if it adds something beyond a total score. Here, the numeric profile label was almost unrelated to the total score, and the main contrast came from how the domains were arranged rather than from a general high-to-low gradient. Figure 1 shows this in two complementary ways. On the original 1-to-10 scale, Profile 1 was relatively higher for information seeking, support, and physician communication, whereas Profile 2 was higher for exercise, household and social activities, symptom management, and emotional management. After centring each participant on their own mean, Profile 1 continued to show relative elevation in information, support, and physician communication; Profile 2 appeared flatter but remained relatively lower in exercise and symptom management. In both configurations, confidence in exercise and symptom management was below confidence in information and physician communication. This may indicate that turning advice into action involves additional physical, emotional, and practical demands, although the present data cannot identify the reason [10,11,12,13,14].

For health and social care, the distinction matters because understanding advice is only the beginning of self-management. A person may know what is recommended and still need help to practise safely, adapt activity to pain or breathlessness, cope with fear or fatigue, involve family, or reach community resources. Nurses and other care professionals can connect these needs across consultations, home life, rehabilitation, social support, and primary-care follow-up. The profiles may therefore prompt a more useful question (what is blocking action today?) rather than repeating information that the person already understands [15,19,20,26,28,29].

These relative differences should not be called clinical strengths or weaknesses without external validation. They may reflect the difficulty of a task, access to support, symptom burden, response style, or the wording of the items. Because the information domain contains only one item, its internal consistency cannot be estimated. Figure 1 is therefore descriptive: it shows where confidence varies within a participant-centred pattern and suggests questions for a tailored conversation. It does not prescribe a profile-based intervention or identify which person will benefit from a particular service.

### 4.3. Covariance Structure, Model Complexity, and Overfitting

The nine domains were correlated; thus, a search restricted to diagonal models might have produced extra profiles simply to reproduce residual associations. The expanded analysis therefore compared diagonal structures (EEI and VVI) with full covariance structures (EEE and VVV). The selected two-profile model came from the full-covariance group, which permits domains to remain related within a profile. This addresses, but does not eliminate, the local-independence concern. Covariance assumptions can alter the solution, and most random starts for the selected model generated warnings; one fitted covariance matrix was also relatively ill-conditioned. A model can therefore look statistically attractive without providing a dependable clinical classification.

The indicators were treated as approximately Gaussian even though the original responses were bounded from 1 to 10. This approach is transparent and compatible with the mclust analysis, but it cannot reproduce every feature of the questionnaire. Moderate bootstrap stability and the absence of a single enumeration-power guarantee mean that the two-profile solution should be viewed as a hypothesis about this sample, not as a validated taxonomy. Comparisons with factor mixture, ordinal, robust, and other distributional models, together with input from clinicians, social care practitioners, and people living with multimorbidity, would help determine which patterns are meaningful in practice [30,31,32,33,34,35,36].

### 4.4. Health Literacy and Healthcare Utilisation Without Causal Claims

The posterior-weighted HLS means were modestly higher in Profile 2 than in Profile 1, but this comparison does not show that health literacy produced the profile pattern. Health literacy may help people use information, while confidence is also shaped by symptoms, resources, social support, and coordination of care [15,16,17,18,19]. The parent study examined an average association; this analysis describes how confidence is arranged within participants. The two findings therefore address different questions and should not be treated as interchangeable.

The utilisation results require similar caution. After adjustment, the clearest contrast was a lower modelled rate of general-practitioner visits for Profile 2; estimates for emergency department visits, admissions, and hospital nights remained compatible with lower and higher use. Profile models did not improve AIC over total-score models; thus, a more detailed profile description was not automatically a better explanation of service use. The six-month counts did not distinguish planned from unplanned, necessary from avoidable, or disease-specific from unrelated contacts. Modal assignment also leaves some classification uncertainty unaccounted for; when class assignment is imperfect, classify-then-analyse comparisons can be biased, which is why BCH and bias-adjusted three-step methods have been proposed [42,43,44,45]. The age and BMI spline checks changed model fit for some outcomes, reminding us that the primary linear form is a modelling choice rather than a biological fact. No formal multiplicity correction was applied. The results should therefore be described as utilisation across profiles or conditional associations, not as evidence that self-efficacy reduced or caused healthcare use [21,22,23,24,25,26,27,28,29]. For health and social-care teams, the more useful question is whether a contact reflects unmet need, appropriate monitoring, fragmented care, or effective support.

### 4.5. Strengths and Limitations

This study has methodological and practical strengths. A documented correction ledger ties the analysis dataset to the original questionnaires, including the verified age and height values. The expanded search considered the profile count and covariance assumptions rather than stopping at the former upper boundary. The report also records failed random starts, eigenvalue diagnostics, profile sizes, entropy, and posterior probabilities, allowing readers to observe the fit and its numerical fragility. Available-item, domain-omission, and bootstrap analyses examine reproducibility from complementary perspectives. Fifth, participant-centred shape diagnostics and explicit HLS missing-data rules protect the substantive interpretation from being driven by a total score or by treating missing answers as negative responses. Finally, nonlinear sensitivity models and adjusted count models connect the profile description to healthcare and social care questions while preserving the distinction between association and causation. The nine-domain design keeps distinctions that a single score would conceal and relates information, communication, symptoms, activity, emotional coping, and social support to the work shared by nurses, physicians, families, rehabilitation, community services, and social care.

Several limitations remain. The cross-sectional design cannot determine whether self-efficacy precedes utilisation, follows worsening health or hospital contact, or changes with the same clinical and social factors. Recruitment from five primary care centres in one municipality limits generalisability to other Albanian regions, people outside regular primary care, and other health systems. Self-reported self-efficacy and utilisation are susceptible to recall and social-desirability bias. The information domain contains one item, and the Gaussian mixture treats bounded scores as approximately continuous. VVV_G2 had moderate bootstrap stability and concentrated numerical warnings, and the selected profiles have not been prospectively validated against function, quality of life, safety, treatment response, or patient priorities. No conventional a priori power calculation was possible for class enumeration; the sample of 459 supports estimation of broad, well-populated patterns but cannot guarantee recovery of the correct number of profiles or stable inference for small groups [35,36]. The five centres were represented by fixed effects, but the small number of centres prevented reliable cluster-robust standard errors. External outcome models were conditional on modal assignment, did not fully propagate classification uncertainty, and used several exploratory outcomes without a formal multiplicity correction. These limits do not invalidate the descriptive pattern, but they define how cautiously it should be used.

### 4.6. Implications and Future Research

The immediate practice implication is to use the domains as conversation prompts rather than as a score-based triage rule. A nurse, physician, social worker, rehabilitation professional, or community health professional can ask whether the person can turn information into action, exercise safely, monitor symptoms, cope emotionally, and draw on family or community support. The answer may lead to different forms of help: teach-back and accessible information for one person, supported practice or rehabilitation for another, a medication or symptom review for another, and social or emotional support for someone else. Figure 1 is therefore a prompt for conversation, not a basis for determining eligibility, restricting services, or justifying more intensive care on its own.

Replication in other Albanian regions and healthcare settings, with a prespecified broader profile range and alternative covariance, ordinal, and robust distributional assumptions, would test generalisability. Longitudinal research can examine whether patterns remain stable, whether people move between configurations, and whether changes in symptoms, function, support, or health literacy precede changes in self-efficacy. Future external-outcome analyses should use classification-error-adjusted distal methods, such as BCH or bias-adjusted three-step or inclusive approaches, and stronger control of clinically important confounding [42,43,44,45]. Larger multicentre samples could support multilevel modelling and more credible precision assessment. Intervention research can then test whether coordinated, domain-specific support improves self-efficacy, function, quality of life, safety, and appropriate healthcare use [15,20,21,22,23,24,25,26,28]. Involving older people, families, and frontline practitioners would help distinguish statistically reproducible patterns from categories that are meaningful in real care.

## 5. Conclusions

In this Albanian primary-care sample, the expanded analysis identified two broad configurations of chronic-disease self-efficacy. The profiles had similar overall confidence but differed in where confidence was relatively concentrated: information, support, and physician communication in one pattern; exercise, household and social activities, symptom management, and emotional management in the other. The participant-centred shape analysis shows why a total score alone would miss this organisation of confidence, while the near-zero total-score correlation shows why the profiles are not simply a severity scale.

The main contribution is a care-oriented way of interpreting self-efficacy. Assessment can look beyond overall confidence and ask which everyday task remains difficult even after relevant information is understood. This question can connect teach-back with rehabilitation, symptom review with medication support, and emotional or social support with coordinated follow-up across primary, community, and social care. The profiles are conversation prompts, not labels, triage categories, or explanations of healthcare use. Their exploratory status is reflected in the moderate bootstrap stability, singular-covariance warnings, Gaussian treatment of bounded indicators, and lack of a single numerical power guarantee. Health literacy and healthcare utilisation were external descriptions or conditional models, not outcomes caused by profile membership. The findings provide a grounded starting point for person-centred care, while wider use depends on replication, longitudinal validation, appropriate handling of classification uncertainty and multiple comparisons, and partnership with the people and professionals who will apply the results.

## Figures and Tables

**Table 1:** Domain-level descriptive statistics and internal-consistency estimates in the strict primary sample.

Domain	Items	Mean (SD)	Median	IQR	Alpha
Exercise	3	4.60 (2.21)	4.33	3.00–6.33	0.926
Obtaining information	1	6.21 (2.52)	6.00	4.00–8.00	N/A
Support	4	5.90 (2.25)	6.00	4.25–7.75	0.839
Communication with physicians	3	6.38 (2.18)	6.67	4.67–8.00	0.874
Disease management	5	5.54 (1.94)	5.60	4.20–7.00	0.922
Household activities	3	5.77 (2.39)	5.67	4.00–7.67	0.944
Social activities	2	5.33 (2.20)	5.00	4.00–7.00	0.878
Symptom management	6	4.99 (2.05)	5.00	3.50–6.50	0.954
Emotional management	6	5.33 (2.08)	5.33	4.00–7.00	0.958

Note: Each domain includes N = 459. SD = standard deviation; IQR = interquartile range. Alpha cannot be calculated for the single-item obtaining-information domain. The full 33-item scale had alpha = 0.974.

**Table 2:** Leading candidates from the expanded strict-sample model search.

Candidate	Structure	G	N	AIC	mclust BIC	ICL	Entropy	Min n	Min APP	Clean Starts
VVV_G2	VVV	2	459	7675.013	−8125.080	−8181.987	0.814	210	0.931	1/51
VVV_G3	VVV	3	459	7471.626	−8148.790	−8211.926	0.856	114	0.889	1/51
VVV_G4	VVV	4	459	7371.971	−8276.233	−8337.190	0.889	13	0.896	1/51
EEE_G7	EEE	7	459	8010.201	−8480.912	−8588.216	0.872	5	0.897	51/51
VVV_G7	VVV	7	459	6961.342	−8546.897	−8581.482	0.951	14	0.941	1/51
VVI_G5	VVI	5	459	8543.308	−8931.439	−8987.181	0.922	45	0.937	see note

Notes: APP = average posterior probability. For mclust BIC, a less negative value indicates better relative fit. “Clean starts” counts warning-free numerical fits among 51 attempts. EEE and VVV permit within-profile domain associations, whereas EEI and VVI use diagonal covariance. Table S2 contains all 40 candidates (G = 1–10 for each structure).

**Table 3:** Profile-assignment quality and participant-centred domain summaries for the two-profile solution.

Profile	n	Proportion	APP	Mean of 9	Exercise Dev.	Information Dev.	Support Dev.	Physician Dev.	Symptoms Dev.
Profile 1	249	0.542	0.961	5.49	−1.12	1.16	0.72	1.34	−0.58
Profile 2	210	0.458	0.931	5.64	−0.57	0.26	0.09	0.42	−0.35

Notes: APP means average posterior probability. Deviations are participant-centred; the full nine-domain deviation table appears in Supplementary Table S3. Profile labels are neutral and are not clinical categories.

**Table 4:** Conditional full negative-binomial utilisation models comparing Profile 2 with Profile 1.

Outcome	Profile 2 IRR	95% CI	*p*	AIC	Observed Zeros	Predicted Zeros	Simulation Band
General practitioner visits	0.809	0.687–0.953	0.011	1926.218	0.133	0.159	0.126–0.194
Emergency department visits	0.874	0.680–1.123	0.293	1453.557	0.420	0.432	0.390–0.477
Hospital admissions	1.268	0.871–1.846	0.215	913.747	0.693	0.695	0.654–0.741
Hospital nights	1.122	0.685–1.838	0.648	1326.560	0.693	0.680	0.641–0.723

Notes: Models adjusted for age, sex, number of chronic diseases, self-rated health, education, residence, health centre, and BMI. IRRs are conditional on modal profile assignment and the selected mixture. Zero-proportion bands are simulation intervals from the fitted negative-binomial models. AIC values for the corresponding full total-score models were 1918.390, 1445.485, 899.466, and 1304.662.

## Data Availability

The de-identified data are available from the corresponding author upon reasonable request, subject to ethical, legal, and privacy restrictions.

## Supplementary Materials

The following supporting information can be downloaded at https://www.mdpi.com/article/10.3390/nursrep16100367/s1, Figure S1. Model fit across the one- to ten-profile solutions in two analytical samples. Table S1. Verified analytical sample flow. Table S2. Expanded strict-sample model search. Table S3. Selected VVV_G2 profile summaries; participant-centred deviations for the selected profiles; and profile quality. Table S4. Pearson correlation matrix for the nine strict self-efficacy domains. Table S5. Sensitivity and stability diagnostics. Table S6. Negative-binomial zero diagnostics and profile contrasts. Table S7. Verified source-questionnaire correction ledger.

## Public Involvement Statement

No public involvement in any aspect of this research.

## Guidelines and Standards Statement

This manuscript was prepared in accordance with the Strengthening the Reporting of Observational Studies in Epidemiology (STROBE) statement [37].

## Abbreviations

The following abbreviations are used in this manuscript:

AIC	Akaike information criterion
APP	Average posterior probability
ARI	Adjusted Rand index
BCH	Bolck–Croon–Hagenaars method for distal outcomes
BIC	Bayesian information criterion
BMI	Body mass index
CI	Confidence interval
CDSES-33	33-item Stanford Chronic Disease Self-Efficacy Scale
ED	Emergency department
EEE	Equal full covariance model
EEI	Equal diagonal covariance model
GP	General practitioner
HLS	Health literacy score
HLS-EU-Q16	16-item European Health Literacy Survey Questionnaire
ICL	Integrated completed likelihood
ID	Identification
IQR	Interquartile range
IRR	Incidence rate ratio
LPA	Latent profile analysis
N/A	Not applicable
SD	Standard deviation
STROBE	Strengthening the Reporting of Observational Studies in Epidemiology
VVI	Profile-specific diagonal covariance model
VVV	Profile-specific full covariance model
