# Supplementary material for: Chronic Diseases Self-Efficacy Profiles and Healthcare Utilisation Patterns Among Older Adults with Multimorbidity in Albania: A Person-Centred Latent Profile Analysis
Source: Nurs Rep. 2026 Oct 16;16(10):367. doi: 10.3390/nursrep16100367 (PMC13708562; doi:10.3390/nursrep16100367)
Supplement: Supplementary file 1 [file nursrep-16-00367-s001.zip › nursrep-4568104-supplementary.pdf]

Supplementary Materials

Article

Chronic Disease Self-Efficacy Profiles and Healthcare Utilisation Patterns Among Older Adults with Multimorbidity in Albania: A Person-Centred Latent Profile Analysis

This supplementary file accompanies the revised first paper and documents the verified data preparation and expanded person-centred analysis of nine chronic-disease self-efficacy domains among older adults with multimorbidity in Vlora Municipality. It provides the analytical sample flow, the complete one- to ten-profile search across four covariance structures, selected-profile summaries, participant-centred shape diagnostics, missing-data and domain-omission sensitivity analyses, bootstrap stability, health-literacy scoring, conditional utilisation checks, and the source-questionnaire correction ledger. Health literacy and healthcare utilisation were external variables rather than profile indicators. The profiles are exploratory descriptions and should not be read as diagnostic categories.

Table S1. Verified analytical sample flow.

Sample definition	n
Parent dataset	464
Eligible age and condition criteria	464
Strict sample before domain eligibility	459
Strict nine-domain primary sample	459
Available-item sensitivity sample	463
Strict HLS-EU-Q16 complete sample	462
Parent participants missing BMI	0

Note: The strict primary sample required complete responses for all nine self-efficacy domains. Available-item scoring used observed responses for the multi-item domains and was used only as a sensitivity analysis. Strict HLS-EU-Q16 scoring required all 16 items; the prorated sensitivity score was calculated only when at least 14 items were valid and was scaled to the 16-item range. An incomplete questionnaire was not converted to zero through `rowSums(..., na.rm = TRUE)`, because missing responses must remain distinguishable from negative responses.

Table S2. Expanded strict-sample model search.

Candidate	G	N	AIC	mclust BIC	ICL	Entropy	Min n	Min APP	Clean starts	Boundary	Small profile
EEE_G1	1	459	8409.06	-8632.03	-8632.03	NA	459	1.000	1	No	No
EEE_G2	2	459	8315.77	-8580.03	-8581.50	0.991	13	0.979	51	No	Yes
EEE_G3	3	459	8223.54	-8529.08	-8530.58	0.994	5	0.980	48	No	Yes
EEE_G4	4	459	8141.30	-8488.14	-8528.74	0.929	5	0.928	51	No	Yes
EEE_G5	5	459	8101.66	-8489.79	-8605.08	0.842	5	0.883	50	No	Yes
EEE_G6	6	459	8053.26	-8482.68	-8595.95	0.856	5	0.883	50	No	Yes

Candidate	G	N	AIC	mclust BIC	ICL	Entropy	Min n	Min APP	Clean starts	Boundary	Small profile
EEE_G7	7	459	8010.20	-8480.91	-8588.22	0.872	5	0.897	51	No	Yes
EEE_G8	8	459	7977.44	-8489.44	-8600.19	0.879	5	0.885	51	No	Yes
EEE_G9	9	459	7942.47	-8495.76	-8592.08	0.892	5	0.878	51	No	Yes
EEE_G10	10	459	7893.89	-8488.47	-8581.44	0.903	5	0.889	51	Yes	Yes
EEI_G1	1	459	11750.26	-11824.58	-11824.58	NA	459	1.000	1	No	No
EEI_G2	2	459	9871.33	-9986.95	-10009.08	0.927	222	0.979	51	No	No
EEI_G3	3	459	9383.17	-9540.08	-9597.30	0.884	109	0.938	51	No	No
EEI_G4	4	459	9060.88	-9259.08	-9307.11	0.921	42	0.953	51	No	No
EEI_G5	5	459	8865.08	-9104.57	-9170.93	0.907	44	0.919	51	No	No
EEI_G6	6	459	8720.23	-9001.00	-9091.02	0.888	38	0.864	51	No	No
EEI_G7	7	459	8595.16	-8917.23	-9001.26	0.902	19	0.887	51	No	Yes
EEI_G8	8	459	8534.84	-8898.19	-8984.75	0.907	7	0.874	51	No	Yes
EEI_G9	9	459	8478.04	-8882.69	-8966.70	0.911	4	0.848	51	No	Yes
EEI_G10	10	459	8406.01	-8851.95	-8943.40	0.903	3	0.876	51	Yes	Yes
VVI_G1	1	459	11750.26	-11824.58	-11824.58	NA	459	1.000	1	No	No
VVI_G2	2	459	9814.89	-9967.66	-9987.39	0.935	207	0.977	1	No	No
VVI_G3	3	459	9415.26	-9646.48	-9681.59	0.917	85	0.952	1	No	No
VVI_G4	4	459	8822.24	-9131.92	-9182.65	0.920	51	0.950	1	No	No
VVI_G5	5	459	8543.31	-8931.44	-8987.18	0.922	45	0.937	1	No	No
VVI_G6	6	459	8390.47	-8857.05	-8912.72	0.925	35	0.897	1	No	No
VVI_G7	7	459	8246.99	-8792.03	-8848.42	0.929	34	0.920	1	No	No
VVI_G8	8	459	8141.53	-8765.01	-8821.65	0.929	25	0.874	1	No	No
VVI_G9	9	459	8095.94	-8797.87	-8851.94	0.939	14	0.909	1	No	Yes
VVI_G10	10	459	7995.31	-8775.70	-8841.34	0.929	12	0.872	1	Yes	Yes
VVV_G1	1	459	8409.06	-8632.03	-8632.03	NA	459	1.000	1	No	No
VVV_G2	2	459	7675.01	-8125.08	-8181.99	0.814	210	0.931	1	No	No
VVV_G3	3	459	7471.63	-8148.79	-8211.93	0.856	114	0.889	1	No	No
VVV_G4	4	459	7371.97	-8276.23	-8337.19	0.889	13	0.896	1	No	Yes
VVV_G5	5	459				NA			0	No	Yes
VVV_G6	6	459				NA			0	No	Yes
VVV_G7	7	459	6961.34	-8546.90	-8581.48	0.951	14	0.941	1	No	Yes
VVV_G8	8	459	7226.61	-9039.26	-9077.73	0.948	18	0.939	1	No	Yes
VVV_G9	9	459	7197.64	-9237.39	-9269.12	0.955	13	0.945	1	No	Yes
VVV_G10	10	459	7218.67	-9485.52	-9515.54	0.954	13	0.945	1	Yes	Yes

Note: The strict search contained 40 candidates: one through ten profiles under each of four covariance structures. Each candidate used one default start plus 50 random starts. Clean starts are warning-free numerical fits among 51 attempts,

and larger (less negative) mclust BIC values are better. VVV_G2 was retained as an exploratory solution after considering relative fit, profile sizes, assignment quality, convergence, covariance diagnostics, parsimony, and interpretability. Numerical warnings and small-profile indicators remain part of the evidence.

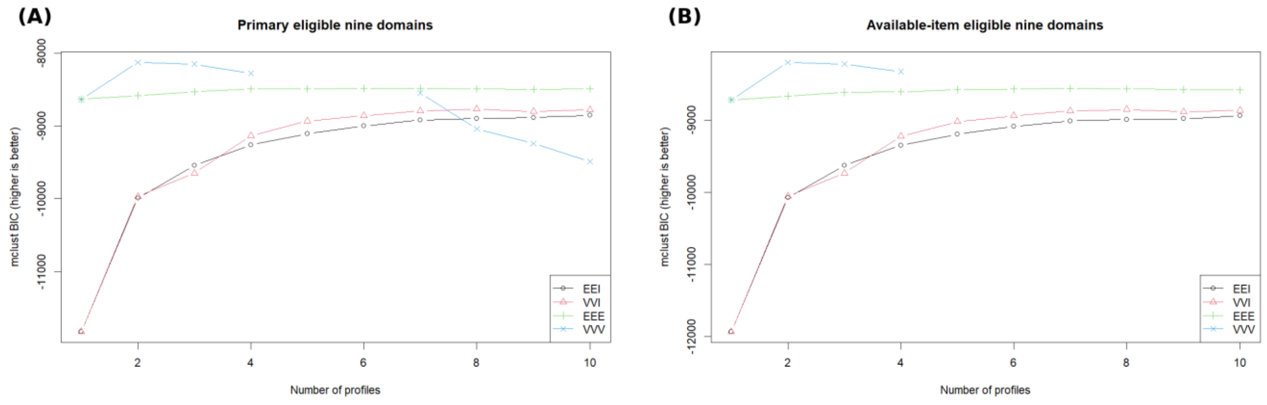

Figure S1. Model fit across the one- to ten-profile solutions in two analytical samples. Panel (A) shows the primary strict nine-domain sample (N = 459); panel (B) shows the available-item sensitivity sample (N = 463). In each panel, larger (less negative) mclust BIC values indicate relatively better fit, but selection also considered profile size, classification quality, numerical diagnostics, parsimony, and interpretability.

Table S3. Selected VVV_G2 profile summaries; Participant-centred deviations for the selected profiles; Profile quality.

Domain	Profile 1 n = 249	Profile 2 n = 210	Profile 1 minus Profile 2
Exercise	4.241	5.021	-0.780
Information	6.526	5.843	+0.683
Support	6.081	5.680	+0.402
Physician	6.701	6.003	+0.698
Management	5.457	5.631	-0.174
Household	5.502	6.078	-0.576
Social	5.028	5.683	-0.655
Symptoms	4.787	5.238	-0.451
Emotional	5.119	5.590	-0.471

Note: Scores range from 1 to 10, with higher scores indicating greater reported confidence. The overall nine-domain means were 5.49 for Profile 1 and 5.64 for Profile 2. Their small difference is consistent with the near-zero correlation between profile assignment and the total 33-item score.

Participant-centred deviations for the selected profiles.

Domain	Profile 1	Profile 2
Exercise	-1.122	-0.566
Information	1.164	0.256

Domain	Profile 1	Profile 2
Support	0.719	0.093
Physician	1.339	0.417
Management	0.094	0.045
Household	0.139	0.491
Social	-0.334	0.097
Symptoms	-0.575	-0.348
Emotional	-0.243	0.003

Participant-centred deviations subtract each participant's own nine-domain mean. Positive values indicate relative elevation and negative values indicate relative attenuation within that person's response pattern. These values are descriptive contrasts, not clinical strengths, deficits, or validated cut-points.

Profile quality.

Profile	n	Proportion	APP
Profile 1	249	0.542	0.961
Profile 2	210	0.458	0.931

Table S4. Pearson correlation matrix for the nine strict self-efficacy domains.

Domain	Exercise	Information	Support	Physician	Management	Household	Social	Symptoms	Emotional
Exercise	1.000	.431	.453	.403	.633	.539	.691	.639	.524
Information	.431	1.000	.637	.730	.582	.504	.450	.488	.467
Support	.453	.637	1.000	.771	.648	.511	.564	.503	.585
Physician	.403	.730	.771	1.000	.680	.594	.528	.532	.578
Management	.633	.582	.648	.680	1.000	.726	.767	.750	.745
Household	.539	.504	.511	.594	.726	1.000	.752	.717	.657
Social	.691	.450	.564	.528	.767	.752	1.000	.792	.710
Symptoms	.639	.488	.503	.532	.750	.717	.792	1.000	.769
Emotional	.524	.467	.585	.578	.745	.657	.710	.769	1.000

Note: $N = 459$. All off-diagonal correlations were positive and $p < 0.001$. These marginal correlations support comparing covariance structures that do and do not impose within-profile local independence, but they do not by themselves determine the correct mixture covariance structure.

Table S5. Sensitivity and stability diagnostics.

Diagnostic	Comparison	Refits or common n	Summary	Interpretation
Available-item versus strict	VVV_G2 versus VVV_G2	458	0.983 adjusted Rand	0.996 ordered agreement
Domain omission	VVV_G2 selected after every omitted domain	9 runs	1 clean start per candidate	Directional sensitivity only
Bootstrap	VVV_G2; 100 refits	100 successful	ARI median 0.584; mean 0.578	Range 0.212-0.865
Random starts	Strict VVV_G2	1/51 warning-free	50 singular-covariance warnings	Positive definite selected fit
Covariance diagnostics	VVV_G2	2 profiles	Min eigenvalues 0.0316 and 0.2340	Condition numbers 252.2 and 18.4

Bootstrap adjusted Rand index values compare refitted classifications of the original participants and are stability diagnostics, not proof of a true latent class structure. Warning-bearing or failed refits were retained in the diagnostic record. The selected VVV_G2 run had 100 successful bootstrap refits; the range of values indicates moderate rather than definitive reproducibility.

Table S6. Negative-binomial zero diagnostics and profile contrasts.

Outcome	Observed zero	Predicted zero	Simulation band	Profile 2 IRR	95% CI	p
GP visits	0.133	0.159	0.126-0.194	0.809	0.687-0.953	0.011
ED visits	0.420	0.432	0.390-0.477	0.874	0.680-1.123	0.293
Hospital admissions	0.693	0.695	0.654-0.741	1.268	0.871-1.846	0.215
Hospital nights	0.693	0.680	0.641-0.723	1.122	0.685-1.838	0.648

All negative-binomial models adjusted for age, sex, number of chronic diseases, self-rated health, education, residence, health centre, and BMI. Observed zero proportions fell within the simulation intervals, so an additional hurdle or zero-inflated component was not required. The profile contrasts are conditional on modal profile assignment and are descriptive or conditional associations, not causal estimates.

Table S7. Verified source-questionnaire correction ledger.

Study ID	Excel row	Variable	Original value	Verified value	Verification status	Source
269	270	Age	37	67	Verified	Author-confirmed original questionnaire
310	311	Height	72	172	Verified	Author-confirmed original questionnaire

The corrections were applied only after checking the source questionnaires and were recorded in the verified rerun ledger: age 37 was corrected to 67 and height 72 was corrected to 172. No automatic range-based replacement was used.
